# Genome-Wide Identification of the Zinc Finger-Homeodomain (*ZF-HD*) Gene Family and Their Response to Cold Stress in *Rosa chinensis*

**DOI:** 10.3390/genes17010090

**Published:** 2026-01-15

**Authors:** Xiaona Su, Yiting Dong, Yuan Liao, Weijian Li, Zheng Chen, Chao Xu, Shaomei Jiang

**Affiliations:** 1 Nanchang Business College, Jiangxi Agricultural University, Jiujiang 332020, China; 2 College of Agriculture, Jiangxi Agricultural University, Nanchang 330045, China; 3 School of Life Sciences, Jiangxi Normal University, Nanchang 330022, China; 4 School of Statistics and Data Science, Jiangxi University of Finance and Economics, Nanchang 330013, China

**Keywords:** *Rosa chinensis*, ZF-HD transcription factor, gene expression, phylogenetic analysis, tissue-specific expression

## Abstract

Background: The zinc finger-homeodomain (ZF-HD) transcription factor family exerts pivotal regulatory functions in plant development and stress responses, yet a systematic genome-wide survey is lacking for *Rosa chinensis*. Methods: In this study, we performed a comprehensive genome-wide identification and analysis of *RcZF-HD* genes in *R. chinensis* using bioinformatics approaches. Nine *RcZF-HD* loci were mined from the rose genome and comprehensively profiled for physicochemical parameters, phylogenetic affiliations, chromosomal positions, exon–intron architectures, conserved motifs, and spatiotemporal expression landscapes. Results: The results showed that *RcZF-HD* genes were unevenly distributed across four chromosomes (Chr2, Chr4, Chr6, and Chr7), with tandem duplication events detected on chromosomes 2 and 7, suggesting their contribution to gene family expansion. Maximum-likelihood phylogeny placed RcZF-HD proteins within nine well-supported sub-clades alongside *Arabidopsis* orthologs, implying both evolutionary conservation and lineage-specific divergence. All members retain canonical zinc-finger domains, yet acquire unique motif signatures predictive of functional specialization. Gene structure analysis revealed considerable diversity in exon–intron organization. Expression profiling across six different tissues (root, stem, leaf, bud, flower, and fruit) demonstrated remarkable tissue-specific expression patterns. Notably, *RchiOBHm_Chr2g0168531* exhibited extremely high expression in stem tissue, while *RchiOBHm_Chr7g0181371* showed preferential expression in flower tissue, suggesting specialized roles in stem development and floral organ formation, respectively. The cold-stress challenge of ‘Old Blush’ petals further disclosed pronounced up-regulation of seven *RcZF-HD* genes, attesting to their critical contribution to low-temperature tolerance. Conclusions: Integrative analyses furnish a multidimensional blueprint of the rose *RcZF-HD* repertoire, providing molecular landmarks for future functional dissection and ornamental trait engineering.

## 1. Introduction

Transcription factors (TFs) are proteins that recognize specific DNA sequences and bind to them, thereby regulating downstream gene transcription initiation through activation or repression. In plants, TFs are deeply involved in the precise control of growth, development, and stress responses, and serve as a central entry point for dissecting gene-regulatory networks. Also, it has been reported that master regulatory TFs exist in plants to regulate the biosynthesis and signaling of these phytohormones [1]. Screening co-binding sites in the promoter region of transcription factors, Virág et al. (2025) found that TF families often share conserved cis-regulatory structures, allowing for coordinated or hierarchical control of gene expression through shared binding motifs [2]. Systematic identification and functional characterization of plant TFs are therefore crucial for elucidating plant life processes and mechanisms of environmental adaptation [3].

ZF-HD (zinc finger-homeodomain) is a plant-specific TF family named after its two core domains: one is the zinc-finger (ZF) domain, usually located at the N-terminus, adopts a C2H2-type zinc-finger fold that mediates DNA binding or protein–protein interactions; the other is the homeodomain (HD) at the C-terminus, ~60 amino acids long, that forms a three-helix structure that binds DNA in a sequence-specific manner and modulates downstream gene expression [4]. ZF-HD proteins are typically divided into two subfamilies: ZHD, which contains both ZF and HD domains; and MIF (mini zinc finger), which possesses only the ZF domain [5,6].

The ZF-HD gene family was first identified in *Arabidopsis thaliana*, where initial studies revealed its involvement in floral development and leaf morphogenesis [4,7]. Subsequent genome-wide identification studies have expanded our understanding of this gene family across various plant species, including rice (*Oryza sativa*), maize (*Zea mays*), and wheat (*Triticum aestivum*) [8,9,10]. These comparative genomic analyses have demonstrated that ZF-HD genes exhibit considerable variation in copy number and structural organization across different plant lineages, suggesting lineage-specific expansion and functional diversification [5,11].

ZF-HD genes have been identified in numerous plant species. Model plants such as *Arabidopsis* and *rice* (*Oryza Sativa* L.) each contain 14–17 members that fall into the ZHD and MIF subgroups [8,12]. In other plants, like *Malus pumila*, *Prunus persica*, *Brassica rapa*, *Camelllia sinensis*, *Zea mays*, *Gossypium hirsutum*, *Triticum aestivum*, and *Rosa chinensis*, their genome-wide surveys have 20, 8, 24, 18, 26, 37, 28, and 9 members, respectively [8,13,14,15,16]. Functional studies show that ZF-HD proteins participate in multiple aspects of plant development and abiotic stress responses: (1) growth and development, e.g., leaf, floral organ, and embryo formation. When the *AtZHD5* in Arabidopsis is overexpressed, the cell size significantly is enlarged, the stem regeneration capacity and the callus proliferation are enhanced, but the root growth is inhibited [17,18]. The GO analysis suggests that the ZHD family may be involved in various metabolic networks within plants, regulating various biological processes and pathways [15]. (2) Abiotic stress tolerance: AtZHD1 can bind to the promoter of the early-response gene ERD1 during plant drought stress, synergistically enhancing plant drought resistance. Plants overexpressing ZHD1 exhibit significantly enhanced drought resistance. In addition, the expression of AtZHD1 can also be induced by high salt and abscisic acid (ABA) [19]. Figueiredo et al. found that four OsZHDs can bind to the promoter of OsDREB1B (DROUGHT RESPONSE ITEM BINDING 1B), inhibiting its transcription and regulating rice’s stress response to drought [20]. In peaches, PpZHD1 interacts with PpOFP1 (OVATE family protein 1) to enhance plant salt tolerance [21]. In wheat, TaZHD is induced to express after salt stress, low temperature, and polyethylene glycol treatment [22]. In chili peppers, CaZHD is induced to express under conditions of high temperature, frost damage, salt stress, and drought [15].

Rose belongs to the species *Rosa rugosa* within the genus Rosa, family Rosaceae, and order Rosales. It is a deciduous shrub with thorny stems and odd-pinnate compound leaves bearing 5–9 oval leaflets with marginal spines. Rose is an important economic crop; its flowers are primarily used for extracting rose essential oil, which is widely employed in cosmetics, food and fine-chemical industries [23]. The optimal temperature for the growth and development of roses is 16–26 °C; when the temperature is below 5 °C, they will enter dormancy [24]. Many studies have reported on plants’ responses to cold stress, including Arabidopsis, rice, wheat, and tomatoes [25,26,27,28]. However, the effects of cold stress on rose plant remain unclear. Furthermore, the lack of a rose genome has kept rose research and industrial application at an early stage, severely constraining their breeding progress. With the rapid development of genomic sequencing and bioinformatics, the first genome of wild *Rosa multiflora* was released in 2018 [29]. Since then, gene-function studies in rose have entered a fast-growing era, and several TF families—including MYB, NAC, bHLH, TCP, and WRKY—have been identified and functionally analyzed [30,31,32,33,34]. However, the ZF-HD family in rose has not yet been investigated.

In the present study, we conducted a systematic genome-wide analysis of the ZF-HD gene family in *R. chinensis*. We identified nine ZF-HD genes and characterized their phylogenetic relationships, chromosomal distribution, gene structure, conserved motifs, and tissue-specific expression patterns. Also, we evaluated the responses of *ZF-HD* genes in ‘Old Blush’ to cold stress. We hypothesized that the *RcZF-HD* repertoire is evolutionarily conserved, yet contains rose-specific duplicates that drive functional partitioning among stems, flowers, and other tissues, and subset members are transcriptionally primed to mediate cold-stress signaling. These hypotheses were tested by integrating genome-wide identification, comparative phylogenomics, and high-resolution expression profiling under both developmental and low-temperature regimes. Our findings provide fundamental insights into the evolutionary conservation and functional diversification of ZF-HD genes in rose, laying the groundwork for future functional studies and potential applications in rose-breeding programs aimed at improving developmental traits and stress tolerance.

## 2. Methods

### 2.1. Identification of ZF-HD Gene Family Members in Rosa chinensis

The genome sequence, protein sequences, CDS sequence, gff3 file, and annotation files of *Malus domestica Golden*, *Prunus mume*, *Arabidopsis thaliana*, *Prunus persica*, *Prunus avium*, and *Rosa chinensis* were downloaded from the EnsemblPlants website (https://plants.ensembl.org/info/data/ftp/index.html) (accessed on 23 February 2025). To identify the RcZF-HD gene family members of the six species, the Hidden Markov Model (HMM) profiles of the zinc finger domain (PF04770) were obtained from the Pfam database (http://pfam.xfam.org/) [35]. HMMER 3.3.2 software (http://hmmer.org/) was employed to search against the *R. chinensis* protein database with an E-value threshold of 1e-5. All candidate ZF-HD proteins were further validated using the NCBI Conserved Domain Database (CDD, https://www.ncbi.nlm.nih.gov/cdd/) (accessed on 14 August 2025) [36] and InterProScan (http://www.ebi.ac.uk/interpro/) (accessed on 14 August 2025) [37] to confirm the presence of both zinc finger and homeodomain structures. Only proteins containing conserved domains were retained as ZF-HD family members. The physicochemical properties of identified ZF-HD proteins were calculated using the ProtParam tool on ExPASy website (https://web.expasy.org/protparam/) (accessed on 26 August 2025) [38]. The obtained protein sequences were analyzed for subcellular localization using the WoLF PSORT online tool (https://wolfpsort.hgc.jp/) (accessed on 27 August 2025).

### 2.2. Phylogenetic Analysis

The ZF-HD protein sequences from *Rosa chinensis* and six species were aligned using the MAFFT software MAFFT (v7.450) (optimization parameters: -maxiterate 1000 -retree 1 -genafpair) with the E-INS-i algorithm. Subsequently, a phylogenetic tree was reconstructed from the resulting multiple sequence alignment using the IQ-TREE (v2.1.4) with a best-fit model according to BIC: JTTDCMut+F+I+R4 (optimization parameters: -m MFP -bb 1000 -alrt 1000 -nt AUTO) with the final inferred phylogenetic tree was visualized and annotated using FigTree software (v1.4.4).

### 2.3. Chromosomal Localization Analysis

To visualize the chromosomal distribution of ZF-HD gene family members in *Rosa chinensis*, the genomic chromosome lengths were first extracted using Samtools (version 1.22, installed via sudo apt install samtools). Subsequently, positional information for all ZF-HD genes was retrieved from the genome annotation file (GFF3 format). Gene identifiers, start/end coordinates, chromosome assignments, and optional color codes were input, and specifications of the MapGene2Chrom website (http://mg2c.iask.in/mg2c_v2.1/) (accessed on 21 October 2025) were used for the generated data visualization.

### 2.4. Gene Structure and Conserved Motif Analysis

The exon–intron organization of ZF-HD genes was analyzed based on the genome annotation file and visualized using the Gene Structure Display Server 2.0 (GSDS, http://gsds.gao-lab.org/) (accessed on 11 September 2025) [39]. Conserved motifs in ZF-HD proteins were identified using the MEME Suite (version 5.3.0) (Multiple Em for Motif Elicitation, https://meme-suite.org/meme/index.html) (accessed on 12 September 2025) [40] with the following parameters: -protein -nostatus -maxsize 600000 -nmotifs 10 -minw 6 -maxw 50, and any number of repetitions (anr) mode.

### 2.5. Three-Dimensional Protein Structure Prediction

The three-dimensional structures of representative ZF-HD proteins were predicted using SWISS-MODEL (https://swissmodel.expasy.org/) website [41] based on homology modeling. The predicted structures were visualized and analyzed using PyMOL software (version 3.1.6.1) (Schrödinger, LLC., New York, NY, USA) to identify the zinc finger and homeodomain regions.

### 2.6. Expression Pattern Analysis

RNA-seq data for different *R. chinensis* tissues from ‘Old Blush’, including root, stem, leaf, ovary, stamen and prickle, were obtained from the NCBI Sequence Read Archive (SRA) database under BioProject accession number PRJNA546486 [30]. Two replicates were sampled for RNA-seq. Then, FastQC software (version 1.12.1) was used to assess the read quality, and Trimmomatic (versioin 0.30) was used to discard the low-quality portions of reads (QUALITY: 15; LEADING: 20; TRAILING: 20; MINLEN: 50; SLIDINGWINDOW: 5: 20) and mapped to the *R. chinensis* reference genome using HISAT2 (v2.1.0) with default parameters [42]. Gene expression levels were quantified as fragments per kilobase of transcript per million mapped reads (FPKM) using StringTie (v2.0.4) [43]. FPKM is a relative quantitative indicator used in transcriptome sequencing to correct gene length and sequencing depth, and it can be used to exhibit gene expression in plants. The expression heatmap was generated using the pheatmap package by python (v3.12.0) with FPKM transformation.

### 2.7. Cold Response of ZF-HD Gene Family in Rosa chinensis

The fresh cut roses from ‘Old Blush’ with consistent growth conditions were randomly divided into two groups, and place them in illuminating incubator. The experiment set up a low-temperature group (4 °C low-temperature treatment), with a normal temperature of 24 °C as the control. The growth condition was set up with a relative humidity control of around 50% and 16 h light/8 h dark. The rose petals were sampled after 5 days of treatment. All the samples were frozen in liquid nitrogen and stored at −80 °C for the following steps.

The RNA of samples was extracted using RNA prep Pure Plant Kit from TOYOBO (TOYOBO Biotech Co., Ltd., Shanghai, China). The first-strand cDNA was synthesized using HiScriptR II Q RT Super Mix (TOYOBO Biotech Co., Ltd., Shanghai, China). The quantitative PCR primers were designed by Beacon designer 7.0 (Supplementary Table S1). Quantitative PCR was conducted in Switzerland using a Roche, Light CyclerR 480 II using SYBR GREEN dye (TaKaRa Biotech Co., Ltd., Dalian, China). The thermos cycle was set as follows: 95 °C for 30 s; 40 cycles of 95 °C for 10 s, and 60 °C for 30 s. b-actin, as a reference gene of Camellia sinensis, was used as an internal control [23]. Quantitative expression analysis in each sample was carried out with each of three biological and technique replicates. Relative gene expressions were analyzed using the 2-ΔCt method. Three biological replicates and three technical replicates were used in this experiment.

## 3. Results

### 3.1. Identification of the ZF-HD Gene Family

To identify ZF-HD genes in rose, a total of nine *RcZF-HD* genes were obtained. The amino acid lengths of these nine ZF-HD proteins range from 86 to 382 amino acids, with molecular weights ranging from 9452.56 to 41,757.38 Da, and isoelectric points (pI) ranging from 6.65 to 8.98. PRQ19624 possesses the longest amino acid sequence, the largest molecular weight, and the smallest pI value. PRQ53619 has the shortest amino acid sequence, the smallest molecular weight, and a pI value of 8.97. PRQ40789 exhibits the highest pI value. Subcellular localization analysis of the RcZF-HD family revealed that PRQ48556, PRQ47599, PRQ53615, PRQ40789, PRQ15836, and PRQ27049 are localized to the nucleus. PRQ53619 and PRQ19624 are localized to the cytoplasm, while PRQ16176 is localized to the chloroplast (Table 1). These findings indicate that there are significant differences in the sequences and characteristics among the nine RcZF-HD proteins.

### 3.2. Phylogenetic Analysis of RcZF-HD Genes

**Figure 1:**
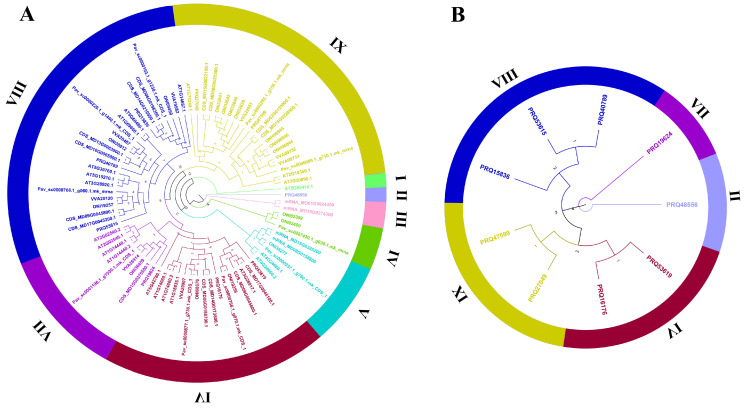
Phylogenetic tree of ZF-HD proteins. (**A**) Phylogenetic tree of roses, containing *Rosa chinensis*, *Malus domestica Golden*, *Prunus mume*, *Arabidopsis thaliana*, *Prunus persica*, *Prunus dulcis*, and *Prunus avium* ZF-HD members. Different-colored arcs represent different groups of ZF-HD proteins. (**B**) ZF-HD proteins phylogenetic tree of *Rosa chinensis*.

The phylogenetic tree was constructed based on gene sequences, elucidating the phylogenetic relationships among genes from multiple species, including *Malus domestica Golden*, *Prunus mume*, *Arabidopsis thaliana*, *Prunus persica*, *Prunus avium*, and *Rosa chinensis*. The phylogenetic analysis classified the genes into nine subclasses (Subclass I to Subclass IX) (Figure 1A). Notably, Subclass I and Subclass II each contain only a single gene, specifically Arabidopsis AT5G65410.1 and rose PRQ48556, respectively. This observation suggests that these two genes diverged early in evolution, exhibiting relatively distant phylogenetic relationships with genes from other subclasses and forming independent evolutionary lineages. Subclass III comprises two genes from golden apple, while Subclass IV clusters two peach genes and one plum gene. Among Subclasses I–IV, only Subclass II includes a rose gene (PRQ48556).

Subclasses V–IX encompass a greater number of genes. Specifically, Subclass VI contains two rose genes (PRQ53619 and PRQ16176), Subclass VII includes one rose gene (PRQ19624), Subclass VIII comprises three rose genes (PRQ53615, PRQ40789, and PRQ15836), and Subclass IX contains two rose genes (PRQ27049 and PRQ47599). Importantly, Subclasses Ⅰ, Ⅲ, Ⅳ, and Ⅴ lack rose genes. The rose genes are predominantly concentrated in subclasses (Ⅵ–Ⅸ) that contain a higher number of ZF-HD members, indicating that the diversification of rose ZF-HD family members occurred relatively late in evolutionary history.

The phylogenetic tree of rose *RcZF-HD* members (Figure 1B) is relatively simple, with the nine *RcZF-HD* gene family members divided into five subclasses (Subclass II, VI, VII, VIII, and IX), which is consistent with the previous multi-species phylogenetic tree.

### 3.3. Chromosomal Distribution of Rose ZF-HD Genes

**Figure 2:**
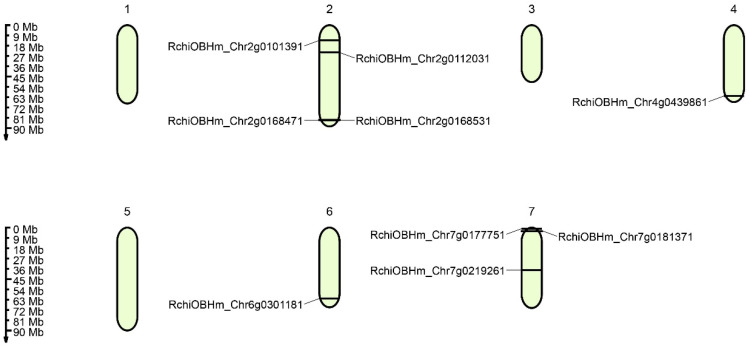
The chromosomal distribution of *RcZF-HD* genes in roses. Chromosomal distribution of *RcZF-HD* genes. *RcZF-HD* gene distribution in the four chromosomes including 2, 4, 6, and 7. Chr2 contains *RcHiOBHm_Chr2g0101391*, *RcHiOBHm_Chr2g0112031*, *RcHiOBHm_Chr2g0168471*, and *RcHiOBHm_Chr2g0168531.* Chr4 has a single *RcZF-HD* gene, *RcHiOBHm_Chr4g0439861*. Chr6 holds *RcHiOBHm_Chr6g0301181*, while Chr7 is home to *RcHiOBHm_Chr7g0177751*, *RcHiOBHm_Chr7g0219261*, and *RcHiOBHm_Chr7g0181371*. The numbers on the left stand for the length of the chromosomes and also provide the locations of these genes on their chromosomes.

The *Rosa chinensis* genes were unevenly distributed on seven chromosomes (Figure 2). Chromosome 1 contained no *RcZF-HD* genes; Chromosome 2 harbored four *RcZF-HD* genes, namely *RchiOBHm_Chr2g0101391*, *RchiOBHm_Chr2g0112031*, *RchiOBHm_Chr2g0168471*, and *RchiOBHm_Chr2g0168531*; Chromosome 3 contained no *RcZF-HD* genes; Chromosome 4 contained one *RcZF-HD* gene (*RchiOBHm_Chr4g0439861*); Chromosome 5 contained no *RcZF-HD* genes; Chromosome 6 contained one *RcZF-HD* gene (*RchiOBHm_Chr6g0301181*); and Chromosome 7 harbored three *RcZF-HD* genes, namely *RchiOBHm_Chr7g0177751*, *RchiOBHm_Chr7g0181371*, and *RchiOBHm_Chr7g0219261*. Nine RcZF-HD genes loci are non-randomly distributed across four of the seven chromosomes of *Rosa chinensis*. Chr2 harbors the largest fraction (44.4%, 4/9 genes), followed by Chr7 (33.3%, 3/9 genes), whereas Chr4 and Chr6 each host a single member (11.1%, 1/9 genes). No RcZF-HD genes were detected on the remaining three chromosomes. There was no indication of large-segment gene duplication events of *RcZF-HD* genes across these seven chromosomes, and the gene distribution presented a scattered pattern in specific chromosomes.

### 3.4. Gene Structure Analysis Rose ZF-HD Genes

**Figure 3:**
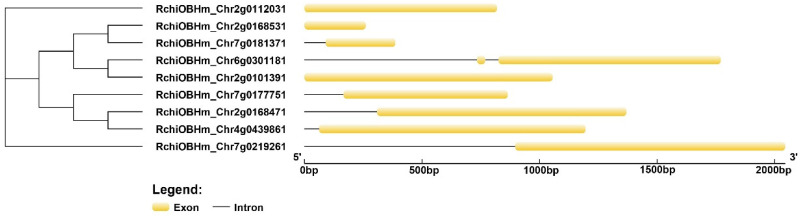
Gene structure of ZF-HD genes. Black lines indicate introns; yellow boxes indicate exons; the genomic length is indicated at the bottom.

To analyze the gene structures of ZF-HD genes, their exon/intron organizations were investigated by comparing genomic sequences (Figure 3). As shown in Figure 3, the *RcZF-HD* gene structures exhibited variations. Most *RcZF-HD* genes were intronless, such as *RchiOBHm_Chr2g0112031*, *RchiOBHm_Chr2g0168531*, and *RchiOBHm_Chr2g0101391*. Notably, only *RchiOBHm_Chr2g0301181* contained two introns, the remaining genes all contained only one intron. Among the nine *RcZF-HD* genes, three loci (*RchiOBHm_Chr2g0101391*, *RchiOBHm_Chr4g0439861*, and *RchiOBHm_Chr6g0301181*) are intronless, corresponding to 33.3% of the family. The remaining six members contain at least one intron, indicating that loss of introns is an architectural feature within the R. chinensis *RcZF-HD* repertoire. The exon/intron distribution patterns showed that genes within the same phylogenetic clade had more similar structures, indicating conserved evolutionary features in their gene architecture.

### 3.5. The Conserved Motif Analysis RcZF-HD Genes

**Figure 4:**
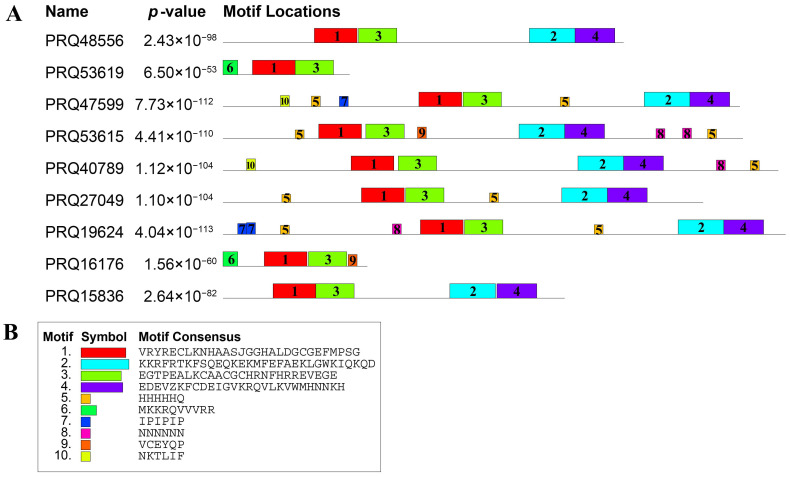
Motif locations and consensus of *RcZF-HD* proteins in roses. Motif composition of ZF-HD proteins. Different colored boxes indicate motifs 1–10. (**A**) Schematic representation of motif locations across *RcZF-HD* proteins. Each row corresponds to a protein, with colored boxes indicating the presence and position of motifs 1–10. (**B**) Motif consensus sequences for *RcZF-HD* proteins. The consensus sequences are provided for motifs 1–10, with each letter representing the most frequently occurring amino acid in that position. *p*-values indicate the statistical significance of motif enrichment in the *RcZF-HD* protein set compared to a random background model. Lower *p*-values suggest higher confidence in motif conservation.

Ten motifs were identified from the nine ZF-HD family members, namely Motif1–10 (as shown in the motif consensus legend) (Figure 4). Motif1 (consensus: VRYRECLKNHAASJGGHALDGCGEEFMPSG) and Motif3 (consensus: EDTEVALKCAACGCHRNKFHRKEVGKE) were present in multiple ZF-HD family members, with Motif1 and Motif3 appearing in PRQ48556, PRQ53619, PRQ47599, PRQ53615, PRQ40789, PRQ27049, PRQ19624, PRQ16176 and PRQ15836. Motif2 (consensus: KKKRPTKFSQPQKEKMFEAEKLGWKIQKQD) and Motif4 (consensus: EDEVZKFCDEIGVKRQVLKVWMHNNKH) was found in PRQ48556, PRQ47599, PRQ53615, PRQ40789, PRQ27049, PRQ19624 and PRQ15836. The ZF-HD family members showed different motif compositions; PRQ47599, PRQ53615, PRQ40789, PRQ27049, and PRQ19624 had relatively more motifs, while PRQ53619 and PRQ16176 had fewer motifs. Motif10 (consensus: NKTLLF) was unique to PRQ47599, PRQ53615, and PRQ40789, and Motif8 (consensus: NNNNNNN) was specific to PRQ53615 and PRQ40789.

### 3.6. Three-Dimensional Structure Prediction of Rose RcZF-HD Genes

**Figure 5:**
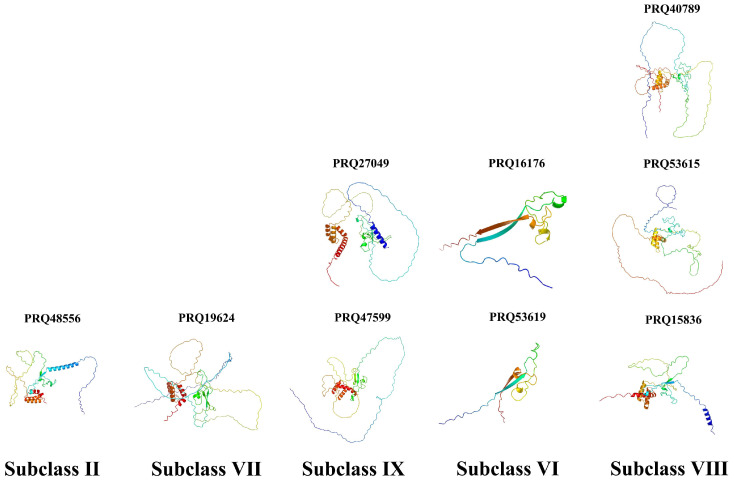
The three-dimensional structure of proteins encoded by 9 *RcZF-HD* transcript factors, which consists of five subgroups (Ⅱ, Ⅵ, Ⅶ, Ⅷ, Ⅸ). The protein structures are displayed in different colors to represent different secondary structure elements.

The three-dimensional structure of *RcZF-HD* family proteins result showed that the three-dimensional structures exhibited notable differences among different *RcZF-HD* members. For instance, PRQ16176 and PRQ53619 displayed structures with prominent β-sheets, while PRQ19624, PRQ27049, PRQ40789, and PRQ48566 contained multiple α-helices, indicating structural diversity within the *RcZF-HD* family (Figure 5).

These 3D models revealed that *RcZF-HD* family proteins had distinct structural features. PRQ15836, PRQ47599, PRQ53615 showed a combination of α-helices and irregular coils, while PRQ16176 and PRQ53619 were dominated by β-sheets. Notably, there were significant structural differences among different *RcZF-HD* members, which might be related to their functional diversification. The structural variations suggested that *RcZF-HD* family proteins have evolved distinct conformations to adapt to different biological roles.

### 3.7. Expression Pattern Analysis of ZF-HD Genes in Rose Tissues

**Figure 6:**
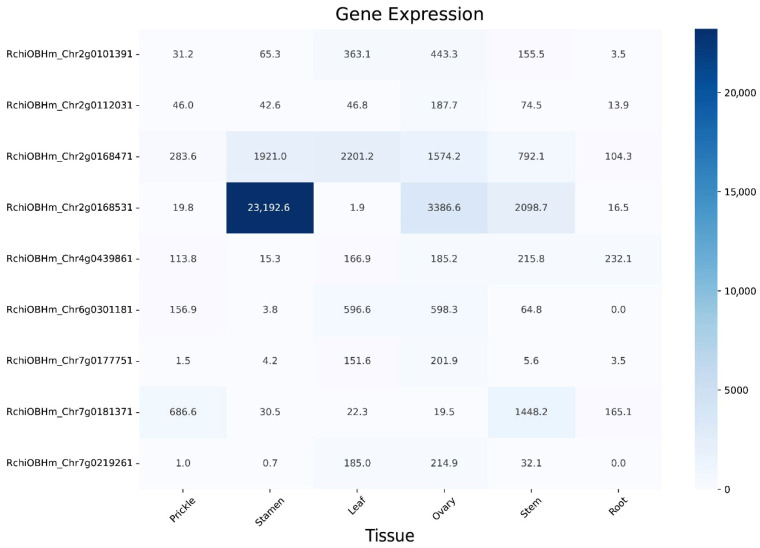
Heat map of *RcZF-HD* gene family members expression data in different rose tissues. The color shade of the dial represents the level of gene expression.

To investigate the specific expression profiles of *RcZF-HD* genes, a gene expression heatmap across different tissues was generated. As shown in the heatmap, the *RcZF-HD* genes exhibited distinct expression patterns in various tissues, including prickle, stamen, leaf, ovary, stem, and root (Figure 6).

Notably, *RchiOBHm_Chr2g0168531* showed an extremely high expression in stamen (FPKM = 23,192.6) and a relatively high expression in ovary (FPKM = 3386.6), indicating a potential crucial role in stamen and ovary development. *RchiOBHm_Chr2g0168531* is highly expressed, it might be associated with processes like pollen development or meiosis, which are crucial for male fertility in plants. Such elevated expression could also signify a response to specific environmental stimuli or developmental cues that are prevalent in the stamen. For instance, genes involved in signaling pathways that regulate pollen grain formation or in the production of proteins that ensure successful fertilization might exhibit high expression levels. This could mean that *RchiOBHm_Chr2g0168531* could be a key transcription factor or regulatory protein that controls a network of genes important for stamen function. *RchiOBHm_Chr2g0168471* was highly expressed in stamen (FPKM = 1921.0), leaf (FPKM = 2201.2), and ovary (FPKM = 1574.2), suggesting its involvement in multiple tissue developmental processes. In contrast, genes like *RchiOBHm_Chr6g0301181* and *RchiOBHm_Chr7g0219261* had an undetectable or very low expression in root, while *RchiOBHm_Chr7g0181371* was highly expressed in prickle (FPKM = 686.6) and stem (FPKM = 1448.2). These expression variations imply that *RcZF-HD* genes may have diverse functions in different tissue development and physiological processes.

### 3.8. The Certification of RNA-Seq Data by qRT-PCR Results

**Figure 7:**
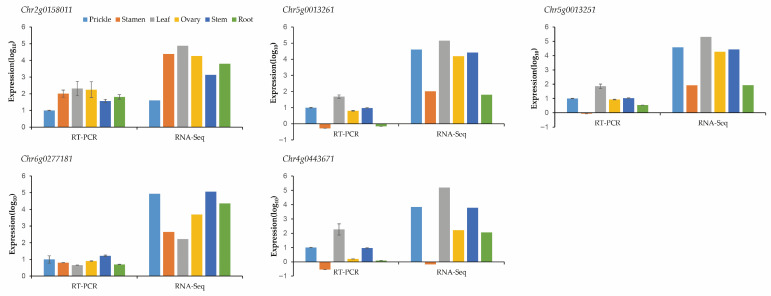
qRT-PCR results confirmed DEG identified by RNA-seq. Five genes were randomly selected for qRT-PCR analysis for validation.

Five genes including *Chr2g0158011*, *Chr5g0013261*, *Chr5g0013251*, *Chr6g0277181*, *Chr4g0443671* were randomly selected for RNA-seq data certification. In general, the qRT-PCR results were basically consistent with the RNA-seq data, suggesting that the RNA-seq data are reliable (Figure 7).

### 3.9. The Response Analysis of ZF-HD Genes in Rose Petal to Cold Stress

**Figure 8:**
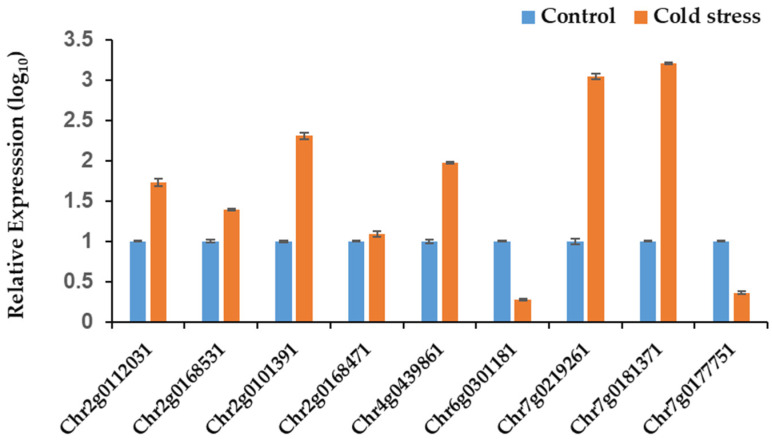
qRT-PCR of *RcZF-HD* gene family members under cold stress in rose petal. Three biological and three technical replicates were used in this experiment.

The qRT-PCR was used to investigate the response of ZF-HD genes in ‘*Old Blush*’ under cold stress in rose after the fresh-cut roses were conducted into cold stress. As shown in Figure 8, seven of the nine detected *RcZF-HD* genes show a violent reaction, especially *RchiOBHm_Chr7g0219261* and *RchiOBHm_Chr7g0181371*, with 110 times and 160 times higher levels in cold stress when compared to the control treatment. However, *RchiOBHm_Chr6g0301181* and *RchiOBHm_Chr7g0177751* exhibited a decrease by about 5 times when compared to the control treatment. The other four genes were shown as changing from 1.22 to 20.12 times. These results indicated their non-negligible roles in cold response in rose.

## 4. Discussion

### 4.1. Evolutionary and Phylogenetic Insights of RcZF-HD Genes

The ZF-HD family within the Rosaceae family, revealing unique evolutionary patterns and late diversification of RcZF-HD genes in *Rosa chinensis*. In this study, nine *RcZF-HD* genes have been identified in *Rosa chinensis* genome through bioinformatics analysis, which is smaller than other plant species, including a total of 11 in *Oryza sativa* [8], 23 in *Triticum aestivum* [10], 14 in *Arabidopsis thaliana* [12], and 21 in *Populus trichocarpa* [44]. The relatively compact *RcZF-HD* gene family in *R. chinensis* may reflect the specific evolutionary trajectory of Rosaceae genomes and their distinct developmental requirements when compared to the model plants and major crops. Phylogenetic analysis of the ZF-HD family from ‘*Malus domestica Golden*’, ‘*Prunus mume*’, ‘*Arabidopsis thaliana*’, ‘*Prunus persica*’, ‘*Prunus avium*’, and rose resolved nine sub-clades in evolutionary (Figure 1). Within the *Rosaceae* family, the divergence in the *RcZF-HD* genes occurred relatively late compared to its divergence in other plant families. This suggests that the RcZF-HD genes in *Rosa chinensis* have undergone a significant degree of evolutionary change more recently than the *Rosaceae* family, potentially adapting to unique ecological niches or developmental processes specific to this lineage. Within the Rosaceae family, the divergence of the RcZF-HD genes occurred relatively late compared to its divergence in other plant families. This is particularly evident in sub-clades III and VI, where the RcZF-HD genes show a higher degree of sequence conservation and a more recent common ancestry compared to genes from *Prunus mume* and *Prunus persica*. In sub-clade I, the RcZF-HD genes exhibit a greater degree of functional specialization compared to *Malus domestica Golden* and *Prunus avium*, suggesting a more recent adaptive radiation within the *Rosaceae* lineage. Furthermore, in sub-clade V, the RcZF-HD genes in *Rosa chinensis* show a distinct evolutionary path compared to *Arabidopsis thaliana*, indicating a unique adaptation to the ecological and developmental requirements of the Rosaceae family. The high sequence identity within each sub-clade implies that they were from a common ancestor, whereas inter-sub-clade divergence reflects species splits.

### 4.2. Gene Structure and Motif Analysis for RcZF-HD Genes in Rosa chinensis

Gene-structure (exon–intron) and motif analyses may illuminate some potential regulatory mechanisms. We uniquely integrate gene structure, protein structure, and motif analysis with phylogenetic classification, offering detailed insights into the correlation between structural features and functional specialization within the RcZF-HD family. Parts of *ZF-HD* genes have no intron, which implies a rapid transcription and functional conservation (Figure 3). The analysis of motifs in Figure 4, in conjunction with the phylogenetic relationships depicted in Figure 1B, shows that universally conserved motifs such as Motif1, Motif2, Motif3, and Motif4 are likely to form the core functional domain essential for the basic function of ZF-HD proteins across species. Sub-family-specific motifs, like Motif10 and Motif8, which are present in certain branches of the phylogenetic tree (Figure 1B), suggest a correlation with functional diversification and evolutionary divergence within the RcZF-HD family. Motif1 Motif2, Motif3 and Motif4 are universally present, likely forming the core functional domain, whereas sub-family-specific motifs (e.g., Motif10, Motif8) denote functional diversification (Figure 4).

Three-dimensional comparisons of PRQ sub-family proteins reveal variable α-helix/β-sheet contents, reinforcing the notion that structural complexity underpins broader biological roles, while conserved regions safeguard core functions (Figure 5). Based on the three-dimensional structural models presented in Figure 5, it is evident that proteins within Subclass II, such as PRQ48556, exhibit a distinct structural arrangement characterized by a notable presence of α-helices, which may be associated with specific regulatory functions. In contrast, Subclass VIII proteins, including PRQ15836, display a more complex structural composition with a higher proportion of β-sheets, potentially indicating a role in structural support or stabilization within the cell. Furthermore, the variability in structural features among subclasses, as observed in the models for PRQ27049 (Subclass IX) and PRQ53619 (Subclass VI), suggests a correlation between protein structure and functional diversity, highlighting the importance of further experimental investigation to elucidate their specific biological roles. The 3D structural model shown in Figure 5 is the prediction result derived from SWISS-MODEL, and it provides a hypothetical explanation for the protein structure.

### 4.3. Tissue-Specific Expression for RcZF-HD Genes

Expression profiling for six rose tissues (prickle, stamen, leaf, ovary, stem, root) revealed their specificity, which provides valuable insights about their potential functional roles in regulating the growth and development (Figure 6). Strikingly, *RchiOBHm_Chr2g0168531* (PRQ53619) reaches 23,192 FPKM in stem, a level 1–3 orders of magnitude higher than in any other organ, strongly implicating this single ZF-HD factor as a master regulator of stem development, putatively controlling vascular-bundle patterning or secondary-wall biosynthesis. It has been reported that rice ZF-HD genes play crucial roles in vascular development. For example, *OsZHD1* and *OsZHD2* regulate vascular bundle formation and internode elongation in rice, directly affecting plant architecture [45,46]. Similarly, in *Populus*, *PagIDD15A* has been shown to regulate xylem differentiation and secondary cell wall biosynthesis [47]. The stem’s highest expression of *RchiOBHm_Chr2g0168531* suggests it may perform analogous functions in rose, making it a promising candidate for manipulating stem characteristics in molecular breeding programs aimed at improving plant architecture. Several other *RcZF-HD* genes exhibited preferential expression in reproductive organs. *RchiOBHm_Chr7g0181371* showed the highest expression in flower tissue (FPKM = 1448.2), suggesting an important role in floral development or flower maturation processes. This observation is consistent with recent discoveries regarding the roles of *ZF-HD* genes in flower development in diverse plant species. Shalmani et al. reported that the *ZF-HD* genes in *Malus pumila* exhibited high expression and might affect flowering process [15]. *AtZHD5* can influence floral structure and leaf formation in *Arabidopsis thaliana* [7]. Wang et al. found that most *BraZF-HD* genes are highly expressed in flowers and play an important role in controlling flowering in Brassica pekinensis [13]. In chrysanthemum, *CmZHD* genes are reported in controlling flower size and ray floret development [48]. A subset of RcZF-HD genes displays near-constitutive expression across all tissues, most notably *RchiOBHm_Chr4g0439861*, implying a housekeeping or regulatory role that underpins fundamental cellular processes. This reveals additional biologically meaningful expression patterns. Conversely, several genes exhibit strict tissue specificity: *RchiOBHm_Chr7g0219261* is almost exclusively expressed in leaf and ovary, while *RchiOBHm_Chr2g0168471* shows a sharp peak in stamen and ovary, suggesting dedicated functions in male and female reproductive development rather than general organ growth. These contrasting profiles underscore a functional division between constitutive regulators and specialized developmental modulators within the RcZF-HD family.

### 4.4. Cold Stress Response for RcZF-HD Genes

Cold stress at 4 °C keeps roses in a good appearance and has a certain preservation effect. Many studies on response to cold stress in plants have been reported. Seven novel Zn-finger TFs were identified bind to the promoter of OsDREB1B via a yeast one-hybrid system screening a cold-induced cDNA expression library. All the Zn-finger TFs identified repressed the of expression OsDREB1B, which plays a key role in cold response in rice [20]. The expression of ZFHD1 was induced by drought, high salinity, and abscisic acid in Arabidopsis. The overexpressed ZFHD1 transgenic plants revealed that several stress-inducible genes were upregulated in Arabidopsis [19]. In this study, under 4 °C cold stress, the *RcZF-HD* genes showed different response expression patterns. Only *Chr6g0301181* and *Chr7g0177751* in rose petals exhibited no significant difference under cold stress, while the other 6 *RcZF-HD* genes showed a significantly upregulated phenotype, signifying a coordinated transcriptional switch that likely enhances membrane stability, antioxidant capacity, or sugar-metabolite accumulation—physiological adjustments previously linked to chilling tolerance in *Rose*. This research represents the first attempt to investigate the cold stress response of the *RcZF-HD* gene family in *Rosa chinensis*, uncovering new aspects of their roles in stress adaptation and development.

Furthermore, the integrating phylogeny, gene structure, motif composition, 3D architecture, and expression data provides a multi-layered portrait of the ZF-HD family. The ZF-HD gene family plays an important role in physiological and biological processes, and the diversity in the expression pattern reflects the adaptive capacity of organisms to environmental changes. This study provides details about the evolutionary and functional characteristics of the ZF-HD gene family in *Rosa chinensis*, and lays a foundation for the future functional characterization of ZF-HD genes. Future studies should expand the phylogenetic breadth and combine molecular genetics to clarify their roles in development and stress responses.

## 5. Conclusions

This study delivers the first comprehensive atlas of the *Rosa chinensis ZF-HD* transcription-factor family, integrating phylogeny, gene structure, motif signatures, 3D protein models, and quantitative expression profiles across tissues and cold stress. Nine *RcZF-HD* genes form a Rosaceae-specific subclass that underwent recent, lineage-restricted diversification, coinciding with the acquisition of intronless architectures and sub-family-exclusive motifs that correlate with structural and functional specialization. Transcriptomic data identify both constitutive regulators and context-specific modulators: *RchiOBHm_Chr2g0168531* (PRQ53619) is a stem-specific master switch (>23,000 FPKM) putatively controlling vascular patterning, whereas *RchiOBHm_Chr7g0181371* (PRQ16176) is restricted to flowers, implying a dedicated role in floral organogenesis. Cold stress elicits a rapid, genome-wide upregulation of six *RcZF-HD* genes, supporting a conserved cold-shield response that may underpin the known preservative effect of 4 °C storage. These multi-layered findings establish RcZF-HD factors as priority targets for mechanistic dissection and precision breeding of rose cultivars with enhanced architectural traits and post-harvest chilling tolerance.

## Figures and Tables

**Table 1:** Detailed information for the ZF-HD gene family in *Rosa chinensis*.

Protein ID	Gene ID	Chromosome Location	Length of CDS (bp)	Number of Amino Acids	Molecular Weight (Da)	Theoretical pI	Predicted Subcellular Localization
PRQ48556	*RchiOBHm_Chr2g0112031*	23797231–23799978	819	272	29,328.24	6.65	Nucleus
PRQ53619	*RchiOBHm_Chr2g0168531*	82900990–82902041	261	86	9452.56	7.60	Cytoplasm
PRQ47599	*RchiOBHm_Chr2g0101391*	12999558–13002515	1056	351	38,824.39	8.89	Nucleus
PRQ53615	*RchiOBHm_Chr2g0168471*	82854849–82856428	1062	353	38,210.93	7.00	Nucleus
PRQ40789	*RchiOBHm_Chr4g0439861*	62189936–62192231	1134	377	41,251.98	8.98	Nucleus
PRQ27049	*RchiOBHm_Chr6g0301181*	61697534–61699875	981	326	35,929.10	6.81	Nucleus
PRQ19624	*RchiOBHm_Chr7g0219261*	37596256–37598657	1149	382	41,757.38	6.95	Cytoplasm
PRQ16176	*RchiOBHm_Chr7g0181371*	3027607–3028180	297	98	10,839.99	8.61	Chloroplast
PRQ15836	*RchiOBHm_Chr7g0177751*	599394–601378	699	232	25,691.69	8.74	Nucleus

## Data Availability

The data presented in this study are openly available in the EnsemblPlants website or other websites, and the RNA-seq data was downloaded from the NCBI Bio-project accession number PRJNA546486.

## Supplementary Materials

The following supporting information can be downloaded at https://www.mdpi.com/article/10.3390/genes17010090/s1, Table S1. Primes used for qRT-PCR.

## References

[1] Virág E., Nagy Á., Tóth B.B., Kutasy B., Pallos J.P., Szigeti Z.M., Máthé C., Kardos G., Hegedűs G. (2024). Master Regulatory Transcription Factors in β-Aminobutyric Acid-Induced Resistance (BABA-IR): A Perspective on Phytohormone Biosynthesis and Signaling in Arabidopsis thaliana and Hordeum vulgare. Int. J. Mol. Sci..

[2] Virág E., Tóth B.B., Kutasy B., Nagy Á., Pákozdi K., Pallos J.P., Kardos G., Hegedűs G. (2025). Promoter Motif Profiling and Binding Site Distribution Analysis of Transcription Factors Predict Autoand Cross-Regulatory Mechanisms in Arabidopsis Flowering Genes. Int. J. Mol. Sci..

[3] Takatsuji H. (1999). Zinc-finger proteins: The classical zinc finger emerges in contemporary plant science. Plant Mol. Biol..

[4] Hong S.Y., Kim O.K., Kim S.G., Yang M.S., Park C.M. (2011). Nuclear import and DNA binding of the ZHD5 transcription factor is modulated by a competitive peptide inhibitor in Arabidopsis. J. Biol. Chem..

[5] Hu W., Ma H. (2006). Characterization of a novel putative zinc finger gene MIF1: Involvement in multiple hormonal regulation of Arabidopsis development. Plant J..

[6] Hu W., Feng B.M., Ma H. (2011). Ectopic expression of the *Arabidopsis* MINI ZINC FINGER1 and MIF3 genes induces shoot meristems on leaf margins. Plant Mol. Biol..

[7] Tan Q.K.G., Irish F.V. (2006). The Arabidopsis zincfinger-homeodomain genes encode proteins with unique biochemical properties that are coordinately expressed during floral development. Plant Physiol..

[8] Jain M., Tyagi A.K., Khurana J.P. (2008). Genome-wide identification, classification, evolutionary expansion and expression analyses of homeobox genes in rice. FEBS J..

[9] Wu L.C., Li M.N., Tian L., Wang S.X., Wu L.J., Ku L.X., Zhang J., Song X.H., Liu H.P., Chen Y.H. (2017). Global transcriptome analysis of the maize (*Zea mays* L.) inbred line 08LF during leaf senescence initiated by pollination-prevention. PLoS ONE.

[10] Liu H., Yang Y., Zhang L.S. (2021). Zinc Finger-Homeodomain transcriptional factors (ZF-HDs) in wheat (*Triticum aestivum* L.): Identification, evolution, expression analysis and response to abiotic stresses. Plants.

[11] Xu G.X., Guo C.C., Shan H.Y., Kong H.Z. (2012). Divergence of duplicate genes in exon-intron structure. Proc. Natl. Acad. Sci. USA.

[12] Hu W., De-Pamphilis C.W., Ma H. (2008). Phylogenetic analysis of the plant-specific Zinc Finger-Homeobox and Mini Zinc Finger gene families. J. Integr. Plant Biol..

[13] Wang W.L., Wu P., Li Y., Hou X.L. (2016). Genome-wide analysis and expression patterns of ZF-HD transcription factors under different developmental tissues and abiotic stresses in Chinese cabbage. Mol. Genet. Genom..

[14] Abdullah M., Cheng X., Cao Y.P., Su X.Q., Manzoor M.A., Gao J.S., Cai Y.P., Lin Y. (2018). Zinc Finger-Homeodomain transcriptional factors (ZHDs) in upland cotton (*Gossypium hirsutum*): Genome-wide identification and expression analysis in fiber development. Front. Genet..

[15] Shalmani A., Muhammad I., Sharif R., Zhao C.P., Ullah U., Zhang D., Jing X.Q., Amin B., Jia P., Tahir M.M. (2019). Zinc Finger-Homeodomain genes: Evolution, functional differentiation, and expression profiling under flowering-related treatments and abiotic stresses in plants. Evol. Bioinform..

[16] Zhou C.Z., Zhu C., Xie S.Y., Weng J.J., Lin Y.L., Lai Z.X., Guo Y.Q. (2021). Genome-wide analysis of zinc finger motif-associated homeodomain (ZF-HD) family genes and their expression profiles under abiotic stresses and phytohormones stimuli in tea plants (*Camellia sinensis*). Sci. Hortic..

[17] Schmidt R., Kunkowska A.B., Schippers J.H. (2016). Role of reactive oxygen species during cell expansion in leaves. Plant Physiol..

[18] Kim J.B., Kang J.Y., Park M.Y., Song M., Kim Y.C., Kim S.Y. (2019). Arabidopsis zinc finger homeodomain protein ZHD5 promotes shoot regeneration and confers other cytokinin-related phenotypes when overexpressed. Plant Cell Tissue Organ Cult..

[19] Tran L.S.P., Nakashima K., Sakuma Y., Osakabe Y., Qin F., Simpson S.D., Maruyama K., Fujita Y., Shinozaki K., Yamaguchi-Shinozaki K. (2007). Co-expression of the stress-inducible zinc finger homeodomain ZFHD1 and NAC transcription factors enhances expression of the ERD1 gene in Arabidopsis. Plant J..

[20] Figueiredo D.D., Barros P.M., Cordeiro A.M., Serra T.S., Lourenco T., Chander S., Oliveira M.M., Saibo N.J. (2012). Seven zinc-finger transcription factors are novel regulators of the stress responsive gene OsDREB1B. J. Exp. Bot..

[21] Tan Q.P., Jiang S., Wang N., Liu X., Zhang X.H., Wen B.B., Fang Y.H., He H.J., Chen X.D., Fu X.L. (2021). OVATE family protein PpOFP1 physically interacts with PpZFHD1 and confers salt tolerance to tomato and yeast. Front. Plant Sci..

[22] Niu H.L., Xia P.L., Hu Y.F., Zhan C., Li Y.T., Gong S.J., Li Y., Ma D.F. (2021). Genome-wide identification of ZF-HD gene family in Triticum aestivum: Molecular evolution mechanism and function analysis. PLoS ONE.

[23] Li W., Geng Z.W., Zhang C.P., Wang K.L., Jiang X.Q. (2021). Whole-genome characterization of *Rosa chinensis* AP2/ERF transcription factors and analysis of negative regulator RcDREB2B in Arabidopsis. BMC Genom..

[24] Giovannini A., Macovei A., Dona M., Valassi A., Caser M., Mansuino A., Ghione G.G., Carbonera D., Scariot V., Balestrazzi A. (2015). Pollen Grain Preservation at Low Temperatures in Valuable Commercial Rose Cultivars. Acta Hortic..

[25] Dai B., Wang H.Y., Li W.Q., Zhang P., Liu T.H., Li X.N. (2024). Ozone Priming Enhanced Low Temperature Tolerance of Wheat (*Triticum aestivum* L.) based on Physiological, Biochemical and Transcriptional Analyses. Plant Cell Physiol..

[26] Kang G.H., Ko Y.J., Lee J.M. (2025). Enhancing virus-mediated genome editing for cultivated tomato through low temperature. Plant Cell Rep..

[27] Liu J.C., Cheng S.R., Yuan X., Chen J.L., Tian M.Q., Zhao Z., Guo T., Xiao W.M. (2025). OsNAL11 and OsBURP12 Affect Rice Seed Germination at Low Temperature. Plant Cell Environ..

[28] Tomás U.L., Victoria B.G., Miguel A.I. (2025). Two antagonistic gene regulatory networks drive Arabidopsis root hair growth at low temperature linked to a low-nutrient environment. New Phytol..

[29] Nakamura N., Hirakawa H., Sato S., Otagaki S., Matsumoto S., Tabata S., Tanaka Y. (2018). Genome structure of Rosa multiflora, a wild ancestor of cultivated roses. DNA Res..

[30] Han Y., Yu J.Y., Zhao T., Cheng T.R., Wang J., Yang W.R., Pan H.T., Zhang Q.X. (2019). Dissecting the Genome-Wide Evolution and Function of R2R3-MYB Transcription Factor Family in *Rosa chinensis*. Genes.

[31] Geng L.F., Su L., Fu L.F., Lin S., Zhang J.M., Liu Q.H., Jiang X.Q. (2022). Genome-wide analysis of the rose (*Rosa chinensis*) NAC family and characterization of RcNAC091. Plant Mol. Biol..

[32] Ding C., Gao J.Z., Zhang S.Y., Jiang N., Su D.T., Huang X.Z., Zhang Z. (2023). The Basic/Helix-Loop-Helix Transcription Factor. Family Gene RcbHLH112 Is a Susceptibility Gene in Gray Mould Resistance of Rose (*Rosa chinensis*). Int. J. Mol. Sci..

[33] Zou Q.C., Dong Q., Tian D.Q., Mao L.H., Cao X.R., Zhu K.Y. (2023). Genome-Wide Analysis of TCP Transcription Factors and Their Expression Pattern Analysis of Rose Plants (*Rosa chinensis*). Curr. Issues Mol. Biol..

[34] Yan X.Y., Zhao J.H., Huang W., Liu C., Hao X., Gao C.Y., Deng M.H., Wen J.F. (2024). Genome-Wide Identification of *WRKY* Transcription Factor Family in Chinese Rose and Response to Drought, Heat, and Salt Stress. Genes.

[35] Mistry J., Chuguransky S., Williams L., Qureshi M., Salazar G.A., Sonnhammer E.L.L., Tosatto S.C.E., Paladin L., Raj S., Richardson L.J. (2021). Pfam: The protein families database in 2021. Nucleic Acids Res..

[36] Lu S.N., Wang J.Y., Chitsaz F., Derbyshire M.K., Geer R.C., Gonzales N.R., Gwadz M., Hurwitz D.I., Marchler G.H., Song J.S. (2020). CDD/SPARCLE: The conserved domain database in 2020. Nucleic Acids Res..

[37] Paysan-Lafosse T., Andreeva A., Blum M., Chuguransky S.R., Grego T., Pinto B.L., Salazar G.A., Bileschi M.L., Llinares-López F., Meng-Papaxanthos L. (2025). The Pfam protein families database: Embracing AI/ML. Nucleic Acids Res..

[38] Garg V.K., Avashthi H., Tiwari A., Jain P.A., Ramkete P.W.R., Kayastha A.M., Singh V.K. (2016). MFPPI–multi FASTA ProtParam interface. Bioinformation.

[39] Hu B., Jin J.P., Guo A.Y., Zhang H., Luo J.C., Gao G. (2015). GSDS 2.0: An upgraded gene feature visualization server. Bioinformatics.

[40] Bailey T.L., Bodén M., Buske F.A., Frith M., Grant C.E., Clementi L., Ren J.Y., Li W.W., Noble W.S. (2009). Meme Suite: Tools for motif discovery and searching. Nucleic Acids Res..

[41] Waterhouse A., Bertoni M., Bienert S., Studer G., Tauriello G., Gumienny R., Heer F.T., de-Beer T.A.P., Rempfer C., Bordoli L. (2018). *SWISS-MODEL*: Homology modelling of protein structures and complexes. Nucleic Acids Res..

[42] Kim D., Paggi J.M., Park C., Bennett C., Salzberg S.L. (2019). Graph-based genome alignment and genotyping with HISAT2 and HISAT-genotype. Nat. Biotechnol..

[43] Pertea M., Pertea G.M., Antonescu C.M., Chang T.C., Mendell J.T., Salzberg S.L. (2015). StringTie enables improved reconstruction of a transcriptome from RNA-seq reads. Nat. Biotechnol..

[44] Hou J., Sun Y., Wang L., Jiang Y.Z., Chen N.N., Tong S.F. (2021). Genome-wide analysis of the Homeobox gene family and identification of drought-responsive members in *Populus trichocarpa*. Plants.

[45] Xu Y., Wang Y.H., Long Q.Z., Huang J.X., Wang Y.L., Zhou K.N., Zheng M., Sun J., Chen H., Chen S.H. (2014). Overexpression of OsZHD1, a zinc finger homeodomain class homeobox transcription factor, induces abaxially curled and drooping leaf in rice. Planta.

[46] Guo M.L., Zheng C., Shi C., Lu X.Z., She Z.Y., Jiang S.Y., Tian D.G., Qin Y. (2025). The OsZHD1 and OsZHD2, Two Zinc Finger Homeobox Transcription Factor, Redundantly Control Grain Size by Influencing Cell Proliferation in Rice. Rice.

[47] Pang H.Y., Dai X.R., Yan X.J., Liu Y.L., Li Q.Z. (2024). C_2_H_2_ zinc finger protein PagIDD15A regulates secondary wall thickening and lignin biosynthesis in poplar. Plant Sci..

[48] Chen C.Y., Yang X.M., Chen S.Y., Chen B., Yue L.R. (2023). Expression Analysis of the ZF-HD Gene Family in *Chrysanthemum nankingense* Under Drought and ABA Treatment. Biotechnol. Bull..

